# Dual Beneficial Effects of (-)-Epigallocatechin-3-Gallate on Levodopa Methylation and Hippocampal Neurodegeneration: *In Vitro* and *In Vivo* Studies

**DOI:** 10.1371/journal.pone.0011951

**Published:** 2010-08-05

**Authors:** Ki Sung Kang, Yujing Wen, Noriko Yamabe, Masayuki Fukui, Stephanie C. Bishop, Bao Ting Zhu

**Affiliations:** Department of Pharmacology, Toxicology and Therapeutics, School of Medicine, University of Kansas Medical Center, Kansas City, Kansas, United States of America; University of Nebraska, United States of America

## Abstract

**Background:**

A combination of levodopa (L-DOPA) and carbidopa is the most commonly-used treatment for symptom management in Parkinson's disease. Studies have shown that concomitant use of a COMT inhibitor is highly beneficial in controlling the wearing-off phenomenon by improving L-DOPA bioavailability as well as brain entry. The present study sought to determine whether (-)-epigallocatechin-3-gallate (EGCG), a common tea polyphenol, can serve as a naturally-occurring COMT inhibitor that also possesses neuroprotective actions.

**Methodology/Principal Findings:**

Using both *in vitro* and *in vivo* models, we investigated the modulating effects of EGCG on L-DOPA methylation as well as on chemically induced oxidative neuronal damage and degeneration. EGCG strongly inhibited human liver COMT-mediated *O*-methylation of L-DOPA in a concentration-dependent manner *in vitro*, with an average *IC*_50_ of 0.36 µM. Oral administration of EGCG moderately lowered the accumulation of 3-*O*-methyldopa in the plasma and striatum of rats treated with L-DOPA + carbidopa. In addition, EGCG also reduced glutamate-induced oxidative cytotoxicity in cultured HT22 mouse hippocampal neuronal cells through inactivation of the nuclear factor κB-signaling pathway. Under *in vivo* conditions, administration of EGCG exerted a strong protective effect against kainic acid-induced oxidative neuronal death in the hippocampus of rats.

**Conclusions/Significance:**

These observations suggest that oral administration of EGCG may have significant beneficial effects in Parkinson's patients treated with L-DOPA and carbidopa by exerting a modest inhibition of L-DOPA methylation plus a strong neuroprotection against oxidative damage and degeneration.

## Introduction

Parkinson's disease (PD) is a chronic and progressive neurological disorder characterized by uncontrolled muscle tremor, rigidity, and bradykinesia. Despite decades of research, at present there is still no cure for the disease. Most of the available therapies will only alleviate the symptoms but will not halt the progression of the disease [1], [2]. Among the currently-used drug treatments for PD, levodopa (L-DOPA), a precursor used in the body for biosynthesis of dopamine, is still considered the most effective drug for relieving motor symptoms. L-DOPA is almost always used in combination with a peripheral dopa decarboxylase inhibitor (such as carbidopa or benserazide), and sometimes a catecholamine-*O*-methyltransferase (COMT) inhibitor (such as tolcapone or entacapone) is also added. While the peripheral dopa decarboxylase inhibitor would effectively prevent the rapid conversion of L-DOPA to dopamine in peripheral tissues, a COMT inhibitor would further prevent it from metabolic conversion (catalyzed by COMT) to form 3-*O*-methyldopa (3-OMD) [3]. Studies have shown that the use of a COMT inhibitor is particularly helpful in controlling the wearing-off phenomenon in PD patients by prolonging the circulating half-life of L-DOPA and improving its brain entry [4]. When this multi-drug combination strategy is used, the effective dose of L-DOPA is reduced, and so are some of the untoward effects that are likely exerted by L-DOPA metabolites, such as dopamine and 3-OMD [5]. Presently, tolcapone and entacapone are the two COMT inhibitors approved for clinical use for enhancement of therapeutic benefits of L-DOPA. However, the use of tolcapone is only limited to fluctuating patients who are refractory to other therapies, and requires heightened monitoring for the occurrence of hepatotoxicity. Although entacapone is relatively safer, it appears less efficacious than tolcapone [6]–[8].

**Figure 1:**
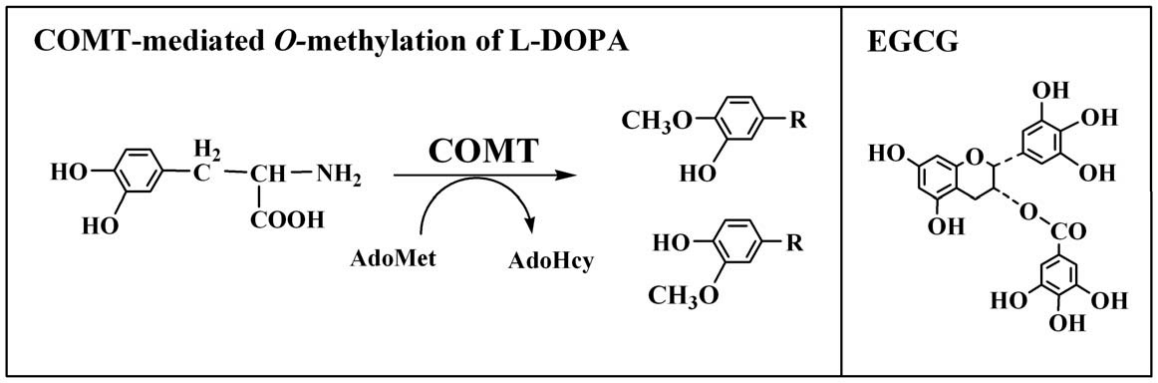
Human COMT-mediated *O*-methylation of L-DOPA (left panel), which results in the formation of two monomethylated products. It is hypothesized that (-)-epigallocatechin-3-gallate (EGCG, structure shown in the right panel) may serve as a naturally-occurring inhibitor of human COMT-mediated *O*-methylation of L-DOPA *in vivo*. In addition, owing to its strong antioxidant activity, it is hypothesized that this tea polyphenol may have additional neuroprotective effects *in vivo*.

In the present study, we sought to determine whether (-)-epigallocatechin-3-gallate (EGCG), a well-known and quantitatively-major tea polyphenol with many health-promoting beneficial effects [9], [10], can serve as a naturally-occurring, safer COMT inhibitor that also possesses neuroprotective actions (see **Figure 1**). This idea was proposed on the basis of the following considerations: First, some of the catechol-containing bioflavonoids and tea catechins are exceptionally good substrates for human COMT [11]. In addition, it was shown previously that bioflavonoids and tea catechins, *e.g.*, (+)-catechin, (-)-epicatechin and EGCG, are also strong inhibitors of the human liver COMT-mediated *O*-methylation of endogenous catechol estrogens [12]. Among these dietary compounds, EGCG was found to be the most potent inhibitor, with an *IC*_50_ of approximately 0.1 µM when 2-hydroxyestradiol was used as substrate [12]. Secondly, oxidative stress and neuronal damage have been considered an important etiological factor in the pathogenesis of PD as well as in the development of adverse effects associated with the long-term use of L-DOPA in PD patients [13], [14]. Tea catechins, especially EGCG, are well-known scavengers of reactive oxygen species (ROS), and they may also function as antioxidants through modulation of transcriptional factors and enzyme activities [9], [15]. It is, therefore, possible that EGCG may be a dietary antioxidant that can be used clinically in PD patients to inhibit COMT-mediated metabolic disposition of L-DOPA while also exerting neuroprotective actions. This intriguing possibility was experimentally examined in the present study. The findings of this study may also shed a mechanistic light on the recent epidemiological observation suggesting that regular tea drinking is associated with a reduced risk of PD [16], [17].

## Results

### *In vitro* inhibition of L-DOPA methylation

**Figure 2:**
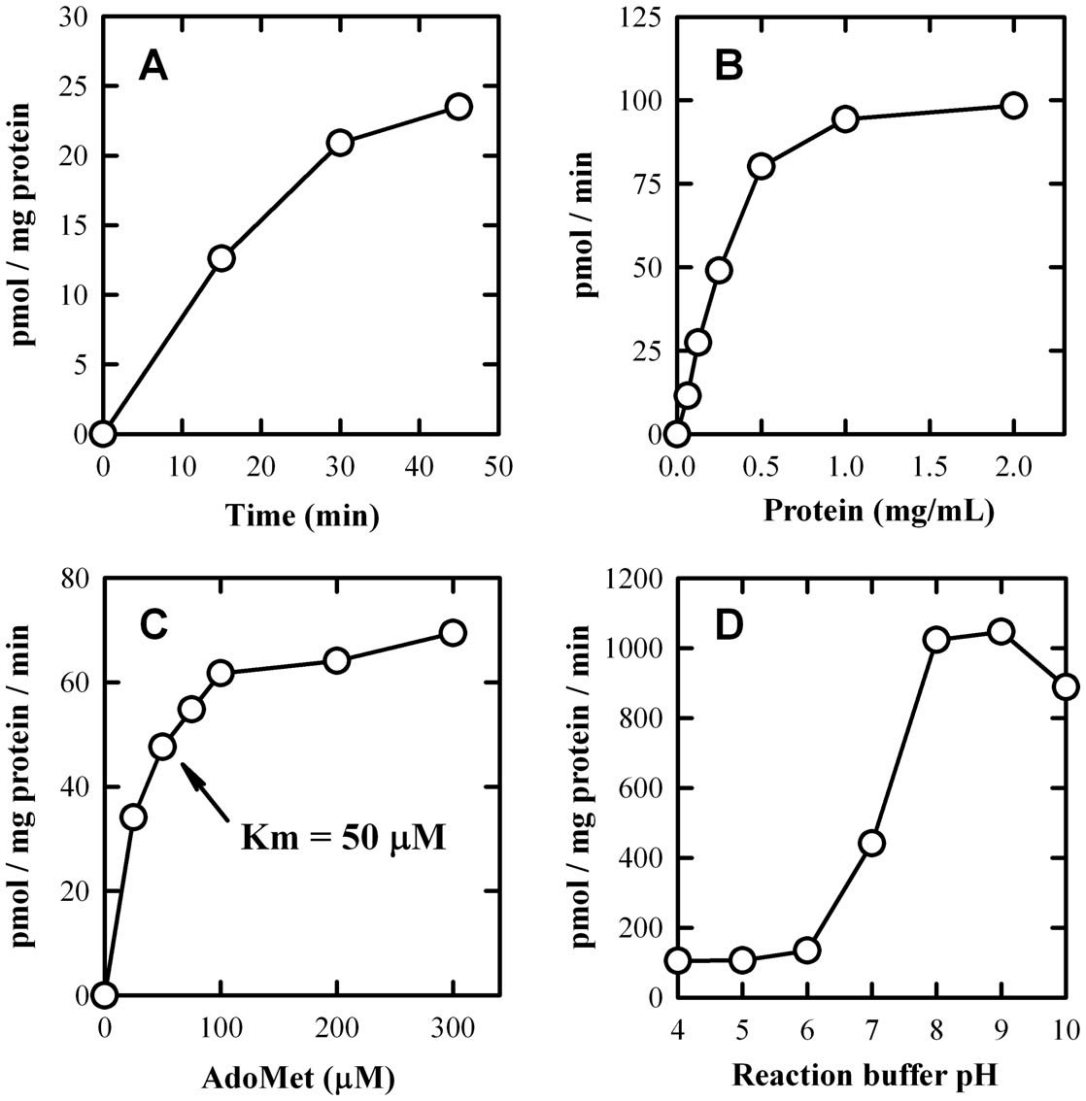
Dependence of the *in vitro O*-methylation of L-DOPA on incubation time (A), cytosolic protein concentration (B), AdoMet concentration (C), and incubation pH (D). The incubation mixture consisted of 10 µM L-DOPA substrate, 250 µM [methyl-^3^H]AdoMet (containing 0.2 µCi) or as indicated (**C**), 0.25 mg/mL of human liver cytosolic protein (HL4C) or as indicated (**B**), 1 mM dithiothreitol, and 1.2 mM MgCl_2_ in a final volume of 1.0 mL Tris-HCl buffer (10 mM) at pH 7.4 or as indicated (**D**). The incubations were carried out at 37°C for 20 min or as indicated (**A**). Each value is the mean of duplicate determinations (with average variations <5%).

We first optimized the reaction conditions for the *in vitro O*-methylation of L-DOPA by determining the effect of variable incubation time and the effect of variable enzyme concentration. The cytosolic COMT prepared from three representative human liver samples (HL4C, HL8C, and HL9C) was used in this study, and a representative data set obtained with HL4C was shown in **Figure 2**. The rate of *O*-methylation of L-DOPA was dependent on the incubation time (linear up to 30 min, **Figure 2A**), cytosolic protein concentration (linear up to 0.5 mg/mL, **Figure 2B**), and [^3^H-methyl]AdoMet concentration (**Figure 2C**). The dependence of *O*-methylation on [^3^H-methyl]AdoMet concentration followed the typical Michaelis-Menten kinetics, with an apparent *K*m value of approximately 50 µM (calculated from the Lineweaver–Burk plot) (**Figure 2C**). We also determined the pH dependence (from pH 4 to 10) for metabolic *O*-methylation of L-DOPA by the cytosol from these three human livers (**Figure 2D**). Based on these measurements, a common reaction condition was devised for COMT-mediated *O*-methylation reactions, which included an incubation time of 10 min, an enzyme concentration of 0.25 mg/mL, a [^3^H-methyl]AdoMet concentration of 250 µM, and a substrate concentration range from 0.1 to 25 µM.

**Figure 3:**
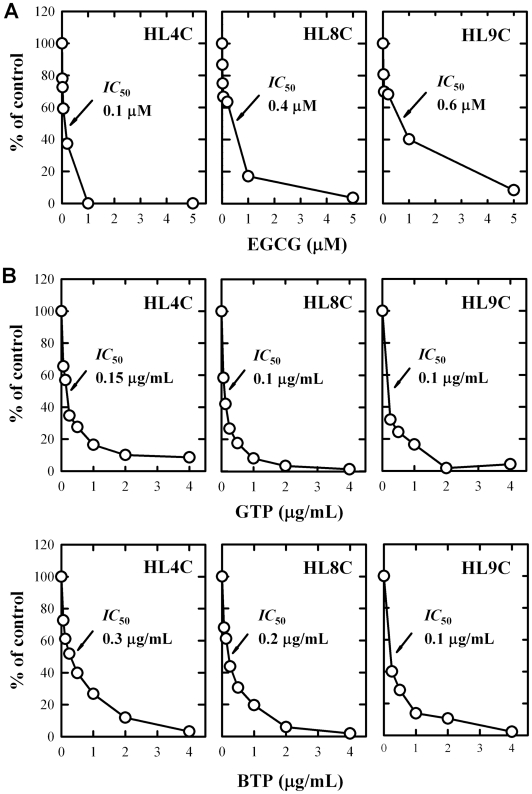
Inhibition of human liver COMT-mediated *O*-methylation of L-DOPA by EGCG, the green tea polyphenol (GTP) extract, and the black tea polyphenol (BTP) extract. The incubation mixture consisted of 10 µM L-DOPA, 250 µM [^3^H-methyl]AdoMet (containing 0.2 µCi), 0.25 mg/mL of human cytosolic protein, 1 mM dithiothreitol, 1.2 mM MgCl_2_, and a dietary inhibitor (concentration as indicated) in a final volume of 0.25 mL Tris-HCl buffer (10 mM, pH 7.4). Incubations were carried out at 37°C for 10 min. Each point is the mean of duplicate determinations (with average variations <5%).

To determine the modulating effect on L-DOPA methylation *in vitro*, the methylation reaction was carried out in the co-presence of varying concentrations of EGCG. EGCG inhibited the COMT-mediated *O*-methylation of L-DOPA in a concentration-dependent manner (**Figure 3A**), with an average *IC*_50_ of 0.36 µM, and a near complete inhibition was seen at 5 µM EGCG. The strong inhibition of L-DOPA *O*-methylation by EGCG was measured in duplicates for each of the human liver cytosolic samples (HL4C, HL8C and HL9C), and consistent results were obtained.

In addition, we have also tested the effect of the green tea polyphenol (GTP) extract and the black tea polyphenol (BTP) extract. Both GTP and BTP extracts strongly inhibited the COMT-mediated *O*-methylation of L-DOPA in a concentration-dependent manner (**Figure 3B**). The GTP extract had a slightly stronger inhibition than the BTP extract, partly due to the presence of higher concentrations of EGCG in the GTP extract than in the BTP extract (43.7% *vs.* 16.2%) [18]. Taken together, these results show that the crude tea extracts and EGCG (a quantitatively-major tea polyphenol) could function as effective inhibitors of human COMT-mediated *O*-methylation of L-DOPA *in vitro*.

### *In vivo* modulation of L-DOPA methylation

**Figure 4:**
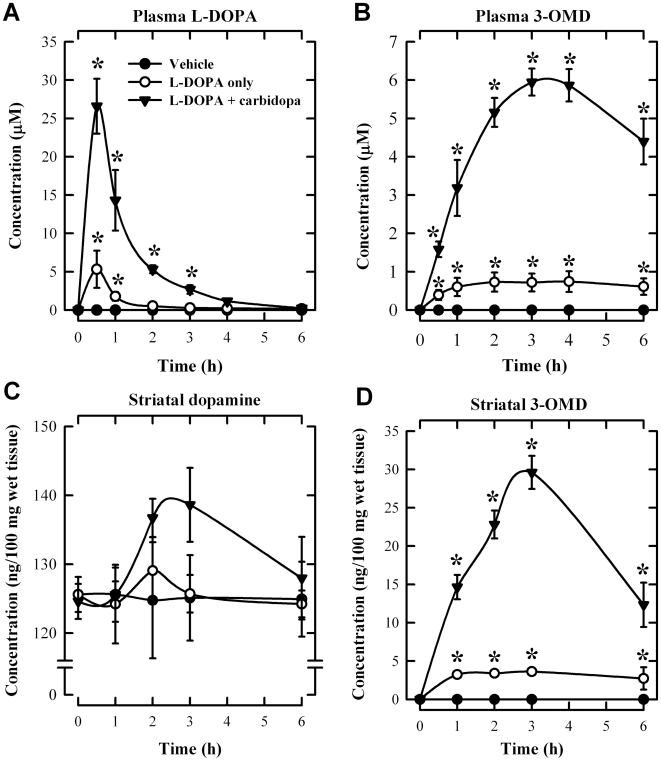
Comparison of the concentrations of the plasma L-DOPA (A), plasma 3-OMD (B), striatal dopamine (C), and striatal 3-OMD (D) in rats. Blood samples were collected from the tail vein at 0.5, 1, 2, 3, 4 and 6 h after oral administration of L-DOPA (20 mg/kg) alone or L-DOPA + carbidopa (20 +5 mg/kg) (N = 3 for each group). The vehicle group received water administration only. Striatal tissues were collected at indicated time points. Vertical bars indicate the standard deviations (S.D.). * *P*<0.05 compared to the vehicle group.

First, we conducted an experiment to determine the suitable doses of L-DOPA + carbidopa for studying L-DOPA methylation *in vivo*. After oral administration of L-DOPA alone (at 20 mg/kg) or L-DOPA + carbidopa (at 20 + 5 mg/kg), there was a rather rapid, short-lasting increase in the plasma L-DOPA level (estimated plasma half-life of approximately 1 h) (**Figure 4A**). In contrast, the level of 3-OMD increased much slower than that of L-DOPA, and it remained elevated for several hours (**Figure 4B**), as reported earlier [19], [20]. The total dopamine level in rat striatum was not increased by treatment with L-DOPA alone but was slightly increased at 2 and 3 h after the combined L-DOPA + carbidopa treatment (**Figure 4C**). Markedly higher striatal 3-OMD level was also observed in animals jointly treated with L-DOPA + carbidopa (**Figure 4D**). Based on these measurements, we chose to use the combined dosing regimen (20 mg/kg L-DOPA +5 mg/kg carbidopa), because L-DOPA methylation under this condition became far more pronounced in both the peripheral compartment and striatum, a situation that would be more suitable for assessing the effect of EGCG on L-DOPA methylation *in vivo*.

**Figure 5:**
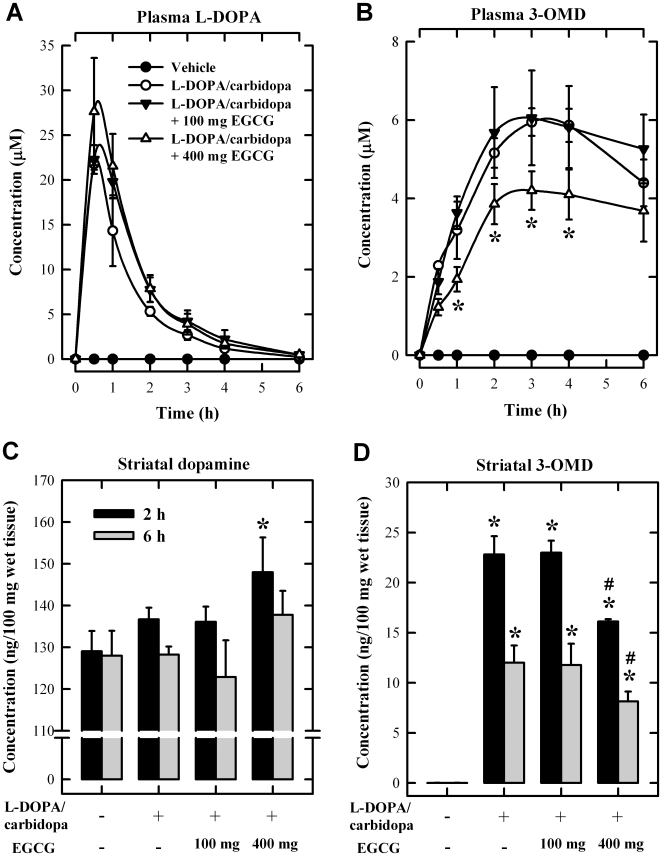
Effect of orally-administered EGCG on the methylation of L-DOPA in rats. **A.** Plasma L-DOPA concentrations. **B.** Plasma 3-OMD concentrations. **C.** Striatal dopamine concentrations. **D.** Striatal 3-OMD concentrations. Rats (N = 3 for each group) were orally administered 100 or 400 mg/kg of EGCG 2 h before oral administration of L-DOPA + carbidopa (20+5 mg/kg). The vehicle group received water administration only. * *P*<0.05 compared to the vehicle treatment group. ^#^ *P*<0.05 compared to the L-DOPA + carbidopa group.

An earlier study showed that EGCG when used at the daily oral dose of up to 500 mg/kg for 13 weeks was not toxic in rats [21]. Similarly, we observed in our pilot experiments that oral administration of EGCG alone at up to 400 mg/kg did not produce any detectable neuronal damage (data not shown). Therefore, we chose to use oral administration of EGCG at 100 and 400 mg/kg in the present study, which was given 2 h before the L-DOPA/carbidopa administration. Under this experimental condition, the plasma L-DOPA level was not significantly affected by oral administration of 100 or 400 mg/kg EGCG (**Figure 5A**), whereas a modest decrease in plasma 3-OMD level was observed in animals treated with 400 mg/kg EGCG, but not in animals treated with 100 mg/kg EGCG (**Figure 5B**). Oral administration of 400 mg/kg EGCG also slightly increased the striatal dopamine level (*P*<0.05) at 2 h after L-DOPA/carbidopa administration, but not at 6 h (**Figure 5C**). The striatal dopamine level was not significantly changed at any time points in rats co-treated with 100 mg/kg EGCG. Similarly, while the striatal 3-OMD level was significantly reduced in animals co-treated with 400 mg/kg EGCG at both 2 and 6 h after L-DOPA/carbidopa administration, its level was not changed in animals co-treated with 100 mg/kg EGCG (**Figure 5D**).

### *In vitro* protection against neuronal oxidative stress and cell death

**Figure 6:**
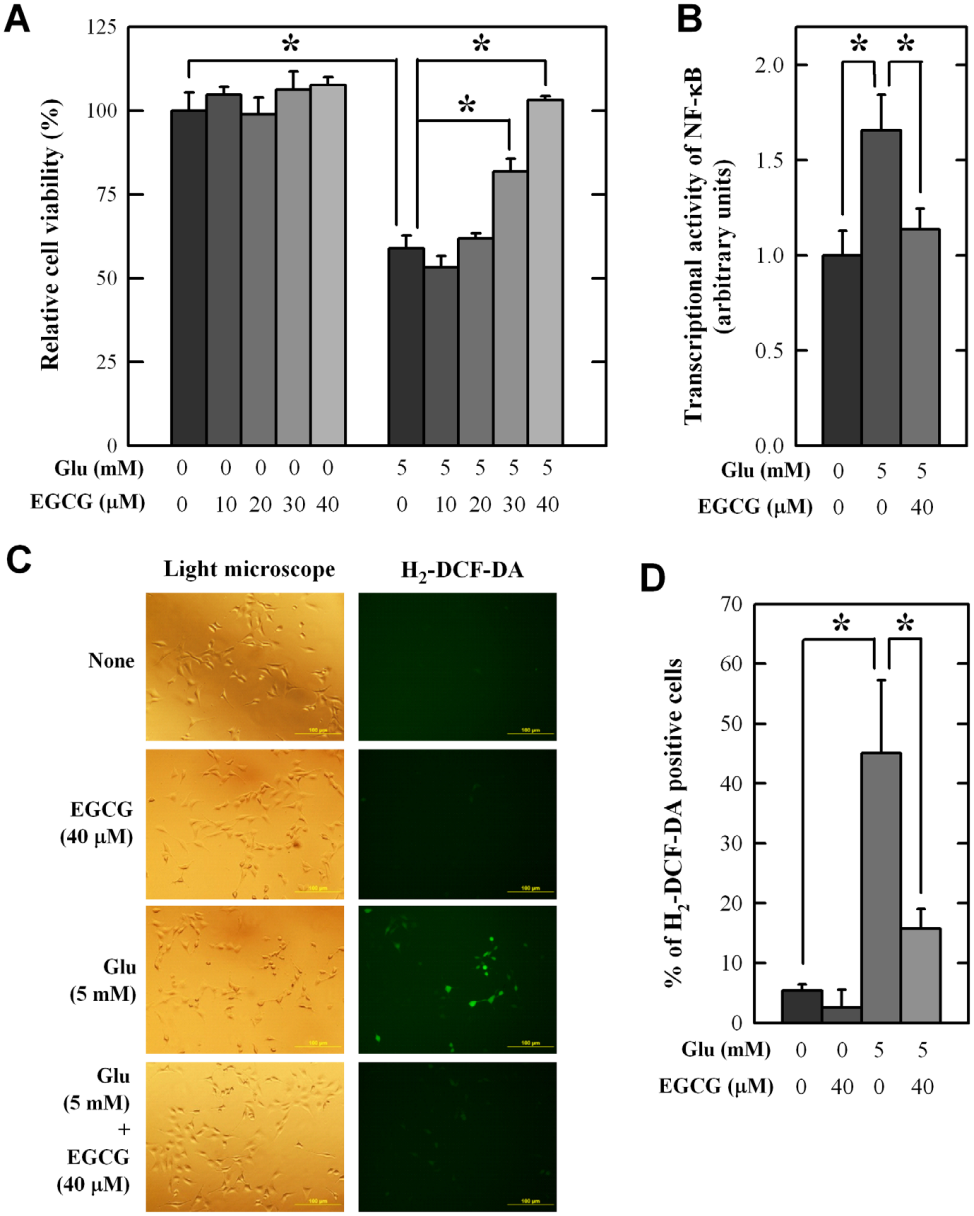
Protective effects of EGCG against glutamate-induced HT22 cell death. **A.** Cell viability. **B.** Transcriptional activity of NF-κB. **C.** ROS generation. **D.** Percentage (%) of H_2_-DCF-DA-positive cells. HT22 cells were treated with 5 mM glutamate and/or EGCG at indicated concentrations. Cell viability, transcriptional activity of NF-κB, NF-κB subcellular localization, and ROS accumulation were determined as described in the Materials and Methods. The experiment was repeated at least 3 times. DAPI staining was employed as a nuclear counter stain. Vertical bars indicate standard deviation (S.D., N = 5). * *P*<0.05 compared to glutamate-treated group.

To investigate the protective effect of EGCG against oxidative stress-associated neuronal cell death, the HT22 cells, an immortalized mouse hippocampal neuronal cell line that is sensitive to glutamate-induced oxidative cytotoxicity [22], was used as an *in vitro* model. As expected, the cell viability was reduced by 40% after 12-h exposure to 5 mM glutamate. Co-treatment of these cells with 5 mM glutamate plus varying concentrations of EGCG reduced glutamate-induced cell death in a concentration-dependent manner (**Figure 6A**). To probe the mechanism of EGCG's neuroprotective effect, we determined the effect of EGCG on the transcriptional activity of NF-κB in HT22 cells treated with glutamate. As shown in **Figure 6B**, the transcriptional activity of NF-κB in HT22 cells transfected with the NF-κB-Luc reporter gene was increased after treatment with 5 mM glutamate for 24 h, but this increase was significantly suppressed by co-treatment with 40 µM EGCG. Notably, our recent study also showed that the intracellular ROS accumulation in glutamate-treated HT22 cells was time-dependent, with a peak occurring at approximately 8 h after glutamate treatment [22]. As shown in **Figure 6C and 6D**, co-treatment of these cells with EGCG strongly suppressed the intracellular ROS accumulation at 8 h after treatment with 5 mM glutamate. Taken together, these data suggest that EGCG's antioxidant activity contributes to the reduction of intracellular ROS accumulation and subsequent NF-κB activation.

### *In vivo* protection against kainic acid-induced neuronal damage

Next, we assessed the neuroprotective effect of orally-administered EGCG *in vivo* using the kainic acid-induced rat hippocampal injury model. The kainic acid-induced selective neuronal damage in rat hippocampus [23] has been a commonly-used *in vivo* model for excitotoxic neuronal damage [24]. Mechanistically, oxidative stress has been identified as a major mechanism for kainic acid-induced hippocampal neurotoxicity in this animal model [25], [26].

EGCG was orally administered 30 min before the intracerebroventricular (i.c.v.) injection of kainic acid (1 µL of the 1 mg/mL solution) into the right lateral ventricle, and the rat was sacrificed 24 h later. All rats that received the i.c.v. injection of kainic acid produced characteristic seizures (such as staring, immobility, and subsequent wet-dog shake), and these effects were generally thought to be due to an over-stimulation of the excitotoxic glutamate receptors by kainic acid. Latency to the first appearance of clearly-defined wet-dog shakes after kainic acid injection was recorded as the seizure latency. In animals receiving both EGCG and kainic acid, no notable differences in latency to the first wet-dog shakes were observed (54.1±9.0 min for rats treated with kainic acid alone *vs.* 48.4±9.1 min for rats treated with EGCG + kainic acid). Here the lack of an appreciable effect of EGCG suggests that this tea polyphenol does not interfere with kainic acid's ability to bind and activate the excitotoxic glutamate receptors in rat hippocampal neurons.

**Figure 7:**
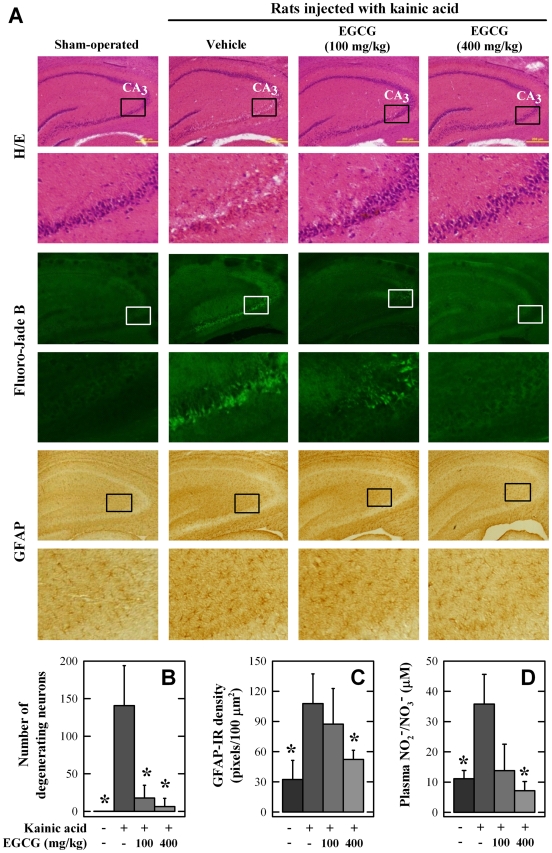
Effect of orally-administered EGCG on kainic acid-induced rat hippocampal injury. **A.** Histopathological and histochemical analyses of the brain tissues. **B.** Number of degenerating neurons based on the Fluoro-Jade B staining. **C.** Expression levels of GFAP-immunoreactive (IR) astrocytes in the CA3 region. **D.** Plasma NO_2_^−^/NO_3_^−^ levels. Rats (N = 3 for each group) were orally administered 100 or 400 mg/kg of EGCG 30 min before kainic acid injection. Kainic acid (1 µL of 1 mg/mL solution) was injected into the right lateral ventricle (anterior/posterior, −1.0; rostral, 1.6; dorsal/ventral, 4.5) using a microliter syringe. * *P*<0.05 compared to kainic acid-treated group.

Additional histological analysis of the damaged brain regions at 24 h post kainic acid injection showed widespread shrinkage of neuronal cell bodies as well as formation of peri-neuronal vacuoles in the CA3 region (H/E staining, **Figure 7A**). In contrast to the CA3 region, the condensation of neuronal cell bodies and vacuolation of neurons in the CA1 region was not observed at 24 h after kainic acid treatment (**Figure 7A**). EGCG treatment significantly reduced the shrinkage of neuronal cells and also the formation of vacuoles in the CA3 region in a dose-dependent manner. The extent of neurodegeneration in the hippocampus was also assessed using the Fluoro-Jade B staining. In kainic acid-treated rats, there was a marked decline in the overall number of neurons as well as an increased number of degenerating neurons in the CA3 region (**Figure 7A, 7B**). In comparison, kainic acid-induced hippocampal neuronal cell death was markedly reduced in EGCG-treated animals (**Figure 7A, 7B**). Similarly, the density of GFAP-positive astrocytes, a parameter that reflects functional changes of astrocytes in damaged regions, was increased following kainic acid injection, but it was markedly reduced in EGCG-treated rats (**Figure 7C**). These data suggest that EGCG may also suppress the early response of hippocampal astrocytes.

To assess whether the antioxidant effect of EGCG is a potential mechanism for its *in vivo* neuroprotective effect, the plasma NO_2_^−^/NO_3_^−^ levels were measured as a marker of oxidative stress. The plasma NO_2_^−^/NO_3_^−^ level was significantly elevated at 24 h after kainic acid injection compared to control animals, but this increase was drastically reduced in animals treated with EGCG (**Figure 7D**).

## Discussion

In the present study, we sought to investigate the beneficial effects of EGCG, a well-known tea polyphenol, on L-DOPA *O*-methylation and oxidative neurodegeneration in an effort to explore the intriguing possibility that certain dietary polyphenols may serve as effective COMT inhibitors as well as neuroprotective agents. We found that EGCG, a quantitatively-abundant polyphenol contained in tea beverages, can strongly inhibit L-DOPA methylation *in vitro* catalyzed by human liver cytosolic COMT (**Figure 3A**), with a high inhibition potency (*IC*_50_ of 0.1–0.6 µM). The molecular mechanism underlying EGCG's high-potency inhibition of human COMT has been recently investigated by using computational molecular modeling approaches [27]. It was suggested that EGCG can bind with a high affinity to the catalytic site of human COMT. Theoretically, EGCG functions as a tight-binding *competitive* COMT inhibitor, but because of the high-affinity nature of its binding to the enzyme, it actually functions like a potent *non-competitive* inhibitor (*i.e.*, functionally similar to an irreversible competitive inhibitor).

When normal rats (treated with L-DOPA + carbidopa) were given an oral administration of EGCG (at 400 mg/kg), their 3-OMD levels in circulation and striatum were reduced by approximately 30%, clearly reflecting an *in vivo* inhibition of L-DOPA methylation. By contrast, only a very small increase (no statistical significance) in L-DOPA plasma concentrations was observed in these animals (**Figure 5**). Notably, our observation of a strong reduction of 3-OMD level but a lack of meaningful increase in L-DOPA plasma concentration in rats treated with L-DOPA + carbidopa + EGCG was similar to the earlier observations with tolcapone or entacapone in rats [28]–[31] or human subjects [32]–[34] that were also treated with L-DOPA + carbidopa. For instance, it was observed that while tolcapone or entacapone at relatively lower doses (3 and 7.5 mg/kg) effectively reduced the circulating and striatal levels of 3-OMD in normal rats co-treated with L-DOPA + carbidopa, their effect on the circulating and striatal L-DOPA levels was much smaller [28], [31], as seen in the present study with EGCG. The reason for the lack of a greater increase in striatal dopamine content in normal rats likely is because its concentration is under a tight regulation to be kept within a narrow normal range. It is possible that in the striatum of PD patients where there is a severe local dopamine deficiency, a greater increase in dopamine concentration likely will be seen when they are co-treated with EGCG. This explanation was supported by an earlier clinical study showing a positive correlation between the degree of striatal dopamine deficiency and the capacity of L-DOPA to increase synaptic dopamine content in PD patients [35].

The observed weaker *in vivo* potency and efficacy of EGCG in inhibiting L-DOPA methylation (based on inhibition of 3-OMD formation) likely are due to its relatively low oral bioavailability in rats (only approximately 2%) [36], [37]. It is known that rodents, in general, have a much higher ability to metabolically dispose phytochemicals than do humans. Because of this reason, the effective doses of EGCG required for PD patients may be considerably smaller than what were used in the present study. Consistent with this suggestion, earlier human studies [38], [39] showed that the circulating concentrations after oral administration of 800 mg EGCG are higher than its effective *IC_50_* concentrations required for inhibiting L-DOPA methylation *in vitro*. Therefore, it is possible that oral administration of EGCG may readily reach therapeutically-effective concentrations needed for inhibiting L-DOPA methylation in PD patients.

An earlier study using ^3^H-EGCG showed that this tea polyphenol can pass through the blood-brain-barrier and are present in the central nervous system [40]. Our observation of a significant reduction in the striatal 3-OMD level in EGCG-treated rats is thought to be due to EGCG's direct inhibition of the central COMT-mediated L-DOPA methylation. It is of interest to point out that the reduction in striatal 3-OMD level may aid in reducing the side effects associated with the long-term L-DOPA/carbidopa therapy. It is known that about 50% of PD patients chronically receiving L-DOPA-based therapy would develop complications within the first 5 years of treatment, which often include severe motor fluctuations (wearing-off phenomenon) and dyskinesia [2], [41], [42]. The exact cause of these adverse effects has not been established at present. 3-OMD, a major metabolite of L-DOPA formed in peripheral and brain tissues, was detected at high levels in the plasma as well as cerebral spinal fluid of PD patients treated with L-DOPA/carbidopa [43], [44], and the plasma levels of 3-OMD in patients with dyskinesia were significantly higher than those from patients without dyskinesia [45]. Mechanistically, it has been suggested that 3-OMD may interfere with the utilization of L-DOPA in the rat brain, and it may also cause neuronal damage via oxidative stress. In addition, it has been suggested that 3-OMD accumulation following long-term L-DOPA administration might contribute to the progression of neurodegeneration in PD patients [13], [46]. Based on these considerations, it is suggested that the ability of EGCG to markedly reduce 3-OMD levels in the periphery and particularly in the striatum would be beneficial for reducing the neuronal toxicity associated with high levels of 3-OMD.

A number of studies have examined the neuroprotective potential of EGCG [47]–[49], which is an antioxidant. It was shown that EGCG can inhibit neuronal damage induced by nitric oxide and β-amyloid [47]–[49]. However, a recent study also reported that EGCG does not protect against 6-hydroxydopamine-induced loss of nigral dopaminergic neurons in rats [50]. In the present study, we sought to further examine the protective effect of EGCG against oxidative degeneration in hippocampal neurons. This brain region is of considerable interest because PD patients are associated with a 6-fold higher risk of developing dementia, a clinical condition characterized by hippocampal atrophy [51], [52]. The HT22 cells, an immortalized mouse hippocampal neuronal cell line, were used in this study as an *in vitro* model. Mechanistically, glutamate induces neurotoxicity in these neuronal cells via inhibition of *N*-acetyl-cysteine uptake, which decreases cellular glutathione levels and ultimately causes oxidative stress and cell death [22], [53], [54]. We found that EGCG, when present at relatively high concentrations (30–40 µM), exerted a strong neuroprotective effect against glutamate-induced oxidative stress in HT22 cells, which is in agreement with an earlier study [55]. To understand the mechanism by which EGCG protects neurons from oxidative damage, we examined its effect on cellular ROS accumulation and NF-κB activation. Earlier studies have shown that cellular oxidative stress caused by ROS accumulation can result in NF-κB activation [56]. We found that EGCG's neuroprotective effect in glutamate-treated HT22 cells is associated with reductions in ROS accumulation and NF-κB transcriptional activity (**Figure 6**). Notably, induction of oxidative stress is also recognized as a major mechanism in kainic acid-induced selective hippocampal damage in rats [23]–[25]. Using this *in vivo* model, therefore, we further demonstrated that a single oral administration of EGCG could effectively reduce kainic acid-induced hippocampal neuronal death (**Figure 7**). In addition, we observed that kainic acid could induce early astrocyte activation in the hippocampal CA3 region, which is in agreement with earlier reports [23], [57]. Co-treatment with EGCG significantly reduced the number of GFAP-positive astrocytes (**Figure 7A**). Collectively, these data suggest that EGCG exerts its *in vivo* protection against chemically-induced neuronal oxidative damage jointly through promotion of neuronal survival and suppression of glial activation. These effects likely will be beneficial for reducing hippocampal impairments in PD patients as well as in reducing neural complications associated with the long-term L-DOPA-based therapy.

It is of considerable interest to note that recent epidemiological studies suggest that regular tea or coffee drinking is associated with a reduced risk of PD [16], [17]. The potential beneficial effects of EGCG (and possibly other tea and coffee polyphenolic compounds) on brain catecholamine metabolism and neuronal survival as observed in this study shed a mechanistic light on these intriguing epidemiological observations. Although the daily intake of EGCG through regular tea drinking may not reach a therapeutically effective concentration, it should be noted that tea and coffee also contain many other polyphenolic antioxidants. The collective neuroprotective effect exerted by these polyphenolic compounds could be significant. Moreover, many of these dietary compounds (such as catechin, quercetin, and caffeic acid) also contain the same catecholic structures as does L-DOPA, which make them good substrates and also effective inhibitors of COMT [11], [12], [18], [27], [36], [58]. Theoretically, a collective inhibition of COMT-mediated *O*-methylation of endogenous catecholamines (including dopamine and L-DOPA) by these dietary polyphenols could be quite significant in the human body. In partial support of this suggestion, it is known that drinking tea and coffee is associated with a number of physiological effects in humans, such as increased heart rate and contractility, increased urination, and strong central nervous system stimulation. These effects are all known to be associated with elevated levels of endogenous catecholamines. While some of these effects associated with tea and coffee often are conveniently attributed to the small amounts of theophylline and caffeine contained in them, the contribution of COMT inhibition has also been suggested in recent years [59], [60].

In summary, we demonstrated in this study that EGCG is an inhibitor of COMT-mediated *O-*methylation of L-DOPA both *in vitro* and *in vivo*. The significant reduction of 3-OMD by EGCG may increase L-DOPA bioavailability in the central nervous system and particularly, reduce potential cytotoxicity associated with elevated levels of 3-OMD. In addition, EGCG also has a strong protective effect against hippocampal neuronal oxidative stress and cell death both *in vitro* and *in vivo*. Taken together, these observations provide an example that some of the dietary polyphenolic compounds may have highly-desirable dual beneficial effects in PD patients that are treated with L-DOPA/carbidopa. Although EGCG *per se* may not be the most ideal dietary compound for this particular therapeutic purpose (in light of its relatively low *in vivo* bioavailability), these findings, nevertheless, provide impetus to search for other dietary polyphenolic compounds (*e.g.*, bioflavonoids and coffee polyphenols) that not only have similar dual beneficial effects in PD but also have more desirable *in vivo* bioavailability. Also, the results from this study raise the possibility for chemically modifying the structure of EGCG to yield safe derivatives that may have markedly improved *in vivo* bioavailability but still retain similar neuronal beneficial effects in PD.

## Materials and Methods

### Chemicals

EGCG, L-DOPA, carbidopa, 3-OMD, dopamine, 3,4-dihydroxybenzylamine hydrobromide (DHBA), glutamate, kainic acid, 2',7'-dichlorofluorescein diacetate, ethylenediaminetetraacetic acid (EDTA) and nitrate reductase were obtained from Sigma Chemical Co. (St. Louis, MO, USA). The black tea polyphenol (BTP) extract and the green tea polyphenol (GTP) extract were gifts from Thomas J. Lipton Company (Englewood Cliffs, NJ). The compositions of the BTP and GTP extracts were described earlier [18]. [^3^H-methyl]AdoMet (specific activity  = 11.2-13.5 Ci mmol^−1^, purity >97%) was purchased from New England Nuclear Research Products (Boston, MA, USA). The plasmid pNF-κB-Luc carrying a firefly luciferase cDNA driven by 5× NF-κB-binding sites was purchased from Stratagene (La Jolla, CA, USA). All other reagents used in this study were obtained from standard suppliers and were of analytical grade (in purity) or better.

### *In vitro* modulation of COMT-catalyzed L-DOPA methylation by EGCG or crude tea extracts

The collection of human liver samples (approved by the Institutional Review Boards (IRBs) of the University of South Carolina, Columbia, SC, and the UMDNJ-Robert Wood Johnson Medical School, New Brunswick, NJ, USA) and the methods for preparation of cytosols from these tissues were described in detail earlier [12]. For assaying the *in vitro O-*methylation of L-DOPA by human liver cytosolic COMT, the incubation mixture usually consisted of 10 µM L-DOPA as substrate, 250 µM [^3^H-methyl]AdoMet (containing 0.2 µCi) as the methyl group donor, 0.25 mg/mL of human liver cytosolic protein as the source of COMT, 1 mM dithiothreitol, 1.2 mM MgCl_2_, and the dietary inhibitor (EGCG, GTP extract, or BTP extract) in a final volume of 0.25 mL Tris-HCl buffer (10 mM, pH 7.4). Incubations were carried out at 37°C for 10 min. The methyl ethers of L-DOPA were measured by using HPLC as described below. Each data point was the mean of duplicate determinations (with average variations <5%).

### Study of the *in vitro* protective effect of EGCG against neuronal oxidative stress and cell death

Glutamate-sensitive HT22 murine hippocampal neuronal cells (a gift from Dr. David Schubert at Salk Institute, La Jolla, CA) were maintained in DMEM supplemented with 10% (*v/v*) FBS and antibiotics (penicillin-streptomycin), and incubated at 37°C under 5% CO_2_. Cells were subcultured once every 2 days. The cells were seeded in 96-well plates at a density of 5000 cells per well. The stock solution of glutamate (1 M in DMEM without serum) and EGCG (20 mM in 100% ethanol) was diluted in the culture medium immediately before addition into each well at desired final concentrations, and the treatment lasted for up to 12 h.

The MTT assay was used for assessment of cell viability as described in our recent study [22]. The relative cell density was expressed as percentage of the control that was not treated with glutamate. For detection of transcriptional activity of NF-κB, HT22 cells in 24-well culture plates were transfected with 0.05 µg pNF-κB-Luc plasmid using Lipofectamine 2000 reagent (Invitrogen, Carlsbad, CA, USA). Twenty-four h after transfection, cells were treated with 5 mM glutamate and/or 40 µM EGCG for 8 h. Then cells were harvested and the luciferase activity was determined by TD-20/20 Luminometer (Turner Designs, Sunnyvale, CA). Firefly luciferase activity was normalized to the protein concentration.

The accumulation of ROS in HT22 cells with or without glutamate treatment was detected using the 2',7'-dichlorofluorescein diacetate (H_2_-DCF-DA) method. H_2_-DCF-DA (1 µM) was added to each well, and incubated for 20 min at 37°C. Then the liquid was removed and PBS was added. Intracellular ROS accumulation was observed and photographed under a fluorescence microscope (AXIO, Carl Zeiss Corporation, Germany).

### Study of the *in vivo* modulation of the *O-*methylation of L-DOPA by EGCG in rats

All procedures involving the use of live animals as described in this study were approved by the Institutional Animal Care and Use Committee (IACUC) of the University of Kansas Medical Center, and strictly followed the guidelines for humane care of animals set forth by the National Institutes of Health. Male Sprague-Dawley rats, weighing 250–270 g, were purchased from Harlan (Indianapolis, IN) and kept in a plastic bottomed cage with a 12-h light/12-h dark cycle, under controlled room temperature (at 25°C) and humidity (60%). They were allowed free access to laboratory pellet chow and water. After arrival, the animals were allowed to acclimate to the new environment for one week before they were used in the experimentation. Rats were randomly divided into various experimental groups (N = 3 to 5) with no significant difference in average body weights. The animals were orally administered 100 or 400 mg/kg EGCG 2 h before administration of L-DOPA and carbidopa (20 and 5 mg/kg, p.o.). Control rats received the same oral administrations of vehicle only. Blood samples were collected from the tail vein with light anesthesia (isoflurane inhalation) at 0.5, 1, 2, 3, 4 and 6 h after L-DOPA + carbidopa administration. About 100 µL blood was drawn, collected in heparin-coated tube, and plasma was obtained from rapid centrifugation and immediately frozen at −80°C until analysis (within 14 days). At 2 and 6 h after blood collection, rats were euthanized with CO_2_ followed by decapitation. The striatal regions of the brain were dissected, weighed, and kept at −80°C until analysis.

Plasma L-DOPA and its metabolites were measured using a method described previously [61] with minor modifications. Plasma samples (50 µL) from each rat were spiked with 5 µL internal standard (DHBA, 100 µM). To precipitate proteins, 20 µL of 1.2 M perchloric acid was added. Tubes were mixed, placed on ice for 10 min, and centrifuged for 4 min at 1250 g at 4°C. Aliquots (30 µL) of the supernatant were added to 60 µL potassium citrate buffer (0.2 M, pH 3.8) to precipitate the perchlorate. Each tube was vortexed for 1 min, left on ice for 10 min, centrifuged for 4 min at 1250 g at 4°C, and then an aliquot of the supernatant was injected onto the HPLC for analysis of composition.

Concentrations of 3-OMD and dopamine in striatum were measured by HPLC with electrochemical detection according to a previously described method [62]. Briefly, the thawed tissues were homogenized using PowerGen 700 (Fisher Scientific, Pitsburgh, PA, USA) in 0.4 M perchloric acid, and the homogenates were centrifuged and filtered for HPLC analysis.

The HPLC system consisted of a Shimadzu pump (LC-10AT model), an electrochemical detector (Coulochem III, ESA Bioscience, Chelmsford, MA, USA), and a HR-80 C18 reverse-phase column (3 µm, 80×4.6 mm, ESA Bioscience). The mobile phase consisted of 50 mM sodium phosphate, 1 mM sodium dodecyl sulfate, 0.67 µL triethylamine, 13.3 µM EDTA, and 8% acetonitrile in water, adjusted to pH 3.0 with phosphoric acid. Before use, the mobile phase was filtered through 0.45 µm (pore size) filter (Millipore, Bedford, MA, USA), and degassed under vacuum. An isocratic elution at a flow rate of 1 mL/min was used.

L-DOPA and its metabolites were identified by comparing their retention times with those of standard compounds. Concentrations were calculated from the peak height with the aid of an internal standard (DHBA). Linearity of the detector responses were tested for all catecholamines, and the coefficients of correlation for all standards were >0.999. The relative standard deviation for the intra-day repeatability was below 5%, representing good precision of the analytical method used.

### Study of the *in vivo* protective effect of EGCG against kainic acid-induced hippocampal injury in rats

In these experiments, male Sprague-Dawley rats were randomly divided into multiple experimental groups (N = 3 to 5 per group), with very similar average body weight for each group. Animals were orally administered 100 or 400 mg/kg EGCG 30 min before kainic acid injection. Kainic acid (1 µL of 1 mg/mL solution) was injected into the right lateral ventricle (anterior/posterior, −1.0; rostral, 1.6; dorsal/ventral, 4.5) using a microliter syringe under anesthesia with ketamine and xylazine (50 and 5 mg/kg, s.c.). The needle was withdrawn 5 min later and scalp was sutured. Experimental control rats were injected with 1 µL of saline instead of kainic acid [63]. Before collecting the brain tissue for analysis (24 h after kainic acid injection), the animals received ketamine and xylazine (50 and 5 mg/kg, s.c.) for anesthesia, and then perfused with 0.1 M neutral phosphate buffered 10% formalin (4% formaldehyde) via the ascending aorta, while the descending aorta was clamped off.

The collected brain tissues were postfixed overnight in the same fixative solution. After cryoprotection in 30% sucrose/phosphate buffer, the tissues were frozen in liquid nitrogen and sectioned serially (30 µm) through the entire brain. The sections were collected in 0.1 M neutral phosphate buffer, mounted on slides, then air-dried on a slide warmer at 50°C for at least half an hour, and stained with hematoxylin and eosin (H/E) for histopathological analysis.

Fluoro-Jade B staining was performed by following the protocols described by Schmued and Hopkins [64] with minor modifications. Briefly, the slides were transferred to a solution of 0.06% potassium permanganate for 10 min, preferably on a shaker to insure consistent background suppression between sections. The staining solution was prepared from a 0.01% stock solution of Fluoro-Jade B that was prepared by adding 10 mg of the dye powder to 100 mL of distilled water. After 20 min in the staining solution, the slides were rinsed and placed on a slide warmer until they were fully dry. The cell bodies of Fluoro-Jade B-positive neurons were viewed under a fluorescence microscope. The number of stained neurons was counted in the obtained images using the Axiovision image analysis software (Carl Zeiss, Inc., Thornwood, NY). For analysis of the hippocampus, the total number of stained neurons was counted in the dorsal hippocampal region (between −1.5 and −2.5 mm from bregma) by using three sections from each rat.

Since astrocyte activation, as evidenced by up-regulation of glial fibrillary acidic protein (GFAP), often was present in damaged neuronal regions, tissue sections were also incubated successively with rabbit monoclonal anti-GFAP antibody (1∶800, Sigma-Aldrich), goat-biotinylated-conjugated polyclonal anti-rabbit antibody (1∶250, Vector Laboratories, CA, USA), and horseradish-peroxidase conjugated avidin-biotin complex (Vector Laboratories). Sections were then exposed to DAB substrate kit (Vector Laboratories) for detection. To perform quantitative analysis of GFAP immunostaining, 3–4 sections per animal were selected and images were captured and analyzed using Axiovision image analysis software. One field (100 µm×100 µm) in each slide within the midpoint of hippocampal CA3 regions was selected for quantification, and the intensity of GFAP immunoreactivity was evaluated according to the relative optical density value.

The plasma nitrite/nitrate (NO_2_^−^/NO_3_^−^) level, a commonly-used ROS index *in vivo*, was determined by converting NO_3_^−^ to NO_2_^−^ with nitrate reductase, followed by addition of Griess reagent for colourimetric measurement of NO_2_^−^ concentrations [65].

### Data analysis

Results were expressed as means ± SD. The statistical significance was determined by analysis of variance (ANOVA) followed by a multiple comparison test with a Bonferroni adjustment. *P* values of less than 0.05 were considered statistically significant.
